# Study on Preparation and Interfacial Transition Zone Microstructure of Red Mud-Yellow Phosphorus Slag-Cement Concrete

**DOI:** 10.3390/ma14112768

**Published:** 2021-05-23

**Authors:** Zhennan Su, Xianhai Li

**Affiliations:** 1 Mining College, Guizhou University, Guiyang 550025, China; zn11Su@163.com; 2 State Key Laboratory of Mineral Processing, Beijing 100160, China; 3 National and Local Joint Laboratory of Engineering for Effective Utilization of Regional Mineral Resources from Karst Areas, Guiyang 550025, China; 4 Guizhou Key Lab of Comprehensive Utilization of Non-metallic Mineral Resources, Guiyang 550025, China

**Keywords:** red mud, yellow phosphorus slag, concrete, mechanical properties, interfacial transition zone, microstructure

## Abstract

Open stockpiling and the continual production of industrial solid wastes such as red mud (RM) and yellow phosphorus slag (YPS) have caused serious environmental pollution issues. Additionally, concrete prepared easily and with high strength is a widely applied building material. Therefore, replacing part or all of the cement for preparing concrete with RM and YPS will greatly reduce this kind of solid waste and, thus, decrease environmental pressures. This study investigated the best ratio for the replacement of concrete with RM and YPS, testing the mechanical properties as well as the morphology, material composition, and microporous structure of the interface transition zone (ITZ). The results showed for the concrete prepared with ordinary Portland cement replaced by 10.00 wt.% RM and 18 wt.% YPS, compared to ordinary Portland cement concrete, the compressive strength of concrete with basalt aggregate and dolomite aggregate increased by 25.04% and 27.27%, respectively, when the concrete was cured with steam for 28 days. Furthermore, it had a smaller average pore diameter and crystal size in the ITZ. The aggregate and matrix were more closely intertwined. This was because RM had a low cementitious activity and mainly had a filling effect when added to concrete, while the highly active silica in YPS could react with the Ca(OH)_2_ crystal (CH) produced from cement hydration to form calcium silicate hydrate (CSH) gel, improving the mechanical properties and microstructure of the concrete.

## 1. Introduction

Bayer red mud (RM) is a byproduct generated when producing alumina (Al_2_O_3_) from bauxite using the Bayer process. However, aluminum products are the most commonly used living materials in various countries, resulting in the generation of a large amount of RM [1]. High-grade bauxite generates about 0.3 tons of RM for every one ton of alumina, but a low-grade version could reach two and one half tons [1]. Furthermore, 72,531 million tons of alumina and 105,169 million tons of RM were produced in China in 2018, but the utilization of RM is below 5% and the quantity of untreated RM in China reached 480–870 million tons [2]. Yellow phosphorus slag (YPS) is a byproduct produced during the production of yellow phosphorus from phosphate minerals using the high temperature electric furnace method. During this process, coke and silica are used as reducing agents and slagging agents, respectively, that react with phosphate minerals at high temperatures. The obtained melting furnace slag is discharged and rapidly cooled by high pressure quenching water, thus forming YPS [3], the chemical formula for which is as follows:

2Ca_3_(PO_4_)_2_ + 6SiO_2_ + 10C ⟶ 6CaSiO_3_ + 10CO + P_4_ (1)

Due to the ratio of the phosphate mineral, the coke and silica contents are different for various production technologies. The YPS composition is also different. Compared to furnace slag, YPS has a similar material composition but lower cementing activity [4,5,6]. Additionally, for one ton of yellow phosphorus, eight to ten tons of YPS are generated. As such, eight million tons of YPS is not treated each year [7]. The massive discharge and accumulation of RM with high alkalinity (pH = 10–13) and YPS including phosphorus (P) and fluorine (F) occupy the land, resulting in the pollution of soil and water, as well as the degradation of environmental protections, impairing resource utilization and sustainable development [3,8]. Therefore, there is an urgent need find a method that utilizes the massive RM and YPS waste to curb environmental pollution.

High-strength concrete is a widely applied building material [9]. Cement is the main component of concrete and also one of the main sources of carbon dioxide. However, carbon dioxide aggravates global warming, and concrete production burns precious fossil fuels [10]. Additionally, with the development of science and technology, as well as improvements in the quality of life, higher requirements are put forward for the strength and durability of concrete. To solve these problems, massive industrial solid wastes e.g., fly ash [11,12,13,14], YPS [15,16], RM [17,18,19,20], blast furnace slag [21,22,23], and steel slag [24,25] as cementitious materials or mineral admixtures to replace part or all of cement in concrete have been studied in recent decades.

Yang et al. [26] ground YPS to two point two microns and then replaced part of the Portland cement with it in a cement mortar. They found that the compressive strength of the ground cement mortar was higher than that of the unground cement. Therefore, the mechanical properties of concrete can be affected by the particle size of the added YPS. Peng et al. [27] found that the mechanical properties and durability of concrete improved, and porosity reduced when adding YPS. Additionally, Zhang et al. [28] studied the hydration mechanisms of cementitious materials mixed with YPS, and the results showed that the early hydration of cement was hindered. Later, the hydration reaction was promoted, which improved the mechanical properties of cement mortar. Besides YPS, RM also made positive effects on cementitious materials or concrete. Yao et al. [29] found that the strength of RM–fly ash as a cementitious material met the requirement for heavy metal leaching. Liu et al. [30] tested the impact of RM on the properties of self-compacting mortar, showing that the leakage of self-compacting mortar was down, and the compressive and flexural strength improved when RM was added. Furthermore, Tang et al. [31] found that the interface transition zone (ITZ) of concrete improved when RM was added.

ITZ is the connecting region between the aggregate and matrix in concrete, the porosity of which is much higher than that of other parts. Importantly, it is the weakest link of concrete due to its high water-binding ratio, as well as its large Ca(OH)_2_ crystal (CH) content, large ettringite (Aft) crystal content, and CH-oriented growth [32]. The mechanical properties of concrete are directly affected by the micro-morphology and ITZ pore structure. Therefore, it is necessary to study the factors affecting ITZ properties. Gao et al. [33] found that ITZ thickness and porosity were influenced by the curing time and the elastic modulus. The adhesive force between the aggregate and paste was affected by the composition and morphology of the aggregate [34]. Additionally, Nežerka et al. [35] found that the ITZ porosity decreased when mineral slags were added. Thus, the replacement cement reached 20 wt.%.

There is plenty of research on RM or YPS applied in concrete and cementitious materials, as shown, yet there is scant research on the influence of both RM and YPS on the mechanical properties and ITZ structure of concrete. The purpose of this study was to use RM and YPS as mineral admixtures when preparing concrete. We also sought to investigate the optimum substitution ratio of cement to consume massive solid wastes and analyze the effects of two kinds of admixtures on the mechanical properties, morphology, material composition, and pore structure of the ITZ in concrete.

## 2. Experimental Procedure

### 2.1. Raw Materials

**Figure 1:**
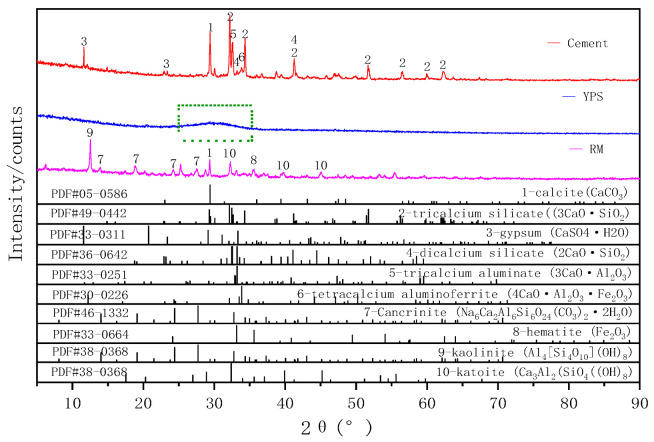
X-ray diffraction (XRD) patterns of red mud (RM), yellow phosphorus slag (YPS), and cement (1: calcite (CaCO_3_), 2: tricalcium silicate (3CaO·SiO_2_), 3: gypsum (CaSO_4_·H_2_O), 4: dicalcium silicate (2CaO·SiO_2_), 5: tricalcium aluminate (3CaO·Al_2_O_3_), 6: tetracalcium aluminoferrite (4CaO·Al_2_O_3_·Fe_2_O_3_), 7: Cancrinite (Na_6_Ca_2_Al_6_Si_6_O_24_(CO_3_)_2_·2H_2_O), 8: hematite (Fe_2_O_3_), 9: kaolinite (Al_4_[Si_4_O_10_](OH)_8_), 10: katoite (Ca_3_Al_2_(SiO_4_((OH)_8_).

The main raw materials used in this study were RM, YPS, cement, and the aggregate. The RM was taken from an alumina refinery in Guizhou, China, which mainly contained cancrinite, katoite, Calcite, clinochlore, kaolinite, hematite, cristobalite, and muscovite, as determined using X-ray diffraction (XRD) (Figure 1). Table 1 shows the contents of SiO_2_, CaO, Fe_2_O_3_, and Al_2_O_3_ in RM, and the results were as follows: 17.33 wt.%, 16.32 wt.%, 21.93 wt.%, and 21.09 wt.%, respectively. The total contents accounted for 76.67 wt.%.

The YPS used in this study was obtained from a phosphorus plant in Guizhou, China. The main chemical components of YPS are SiO_2_ and CaO, which account for 37.22 wt.% and 44.77 wt.%, respectively. Al_2_O_3_, MgO, and K_2_O also were contained in small amounts. XRD showed that YPS has a complex crystal phase and a significantly wider peak in the range of 25–35°, indicating a higher content of amorphous components in YPS and a lower diffraction peak of crystalline minerals, which shows that YPS has a higher potential for cementitious activity.

**Figure 2:**
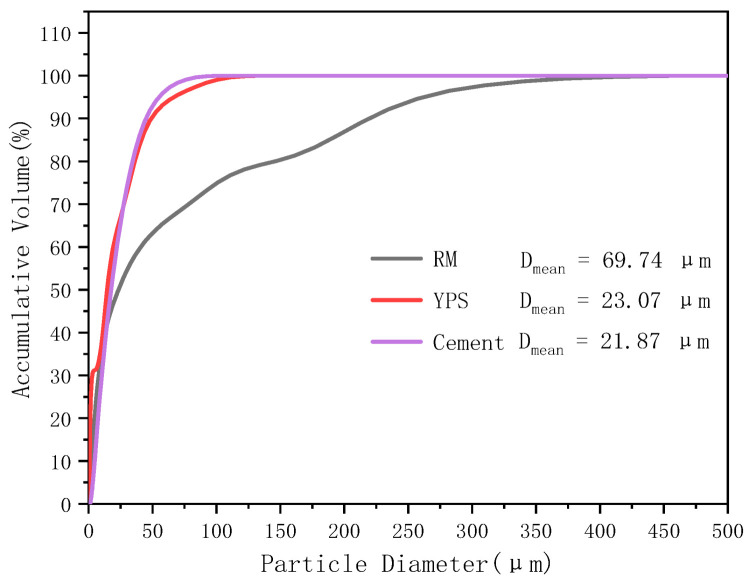
Cumulative particle size distribution of the raw materials.

The cement used in this paper was ordinary Portland cement, which was purchased from the Guizhou Huishui Southwest Cement limited company in Huishui County, Guizhou Province, China. The main minerals in the cement were tricalcium silicate (C_3_S), dicalcium silicate (C_2_S), tricalcium aluminate (C_3_A), and tetracalcium aluminoferrite (C_4_AF). Additionally, the accumulative particle size distribution curves of the RM, YPS, and cement, as determined using a LS13320 laser particle size analyzer (LS13320, Beckman Coulter, Inc, CA, USA), are shown in Figure 2. The result indicated that the YPS particle size was close to that of the cement, but the RM grain size was coarser than that of the cement and YPS.

**Figure 3:**
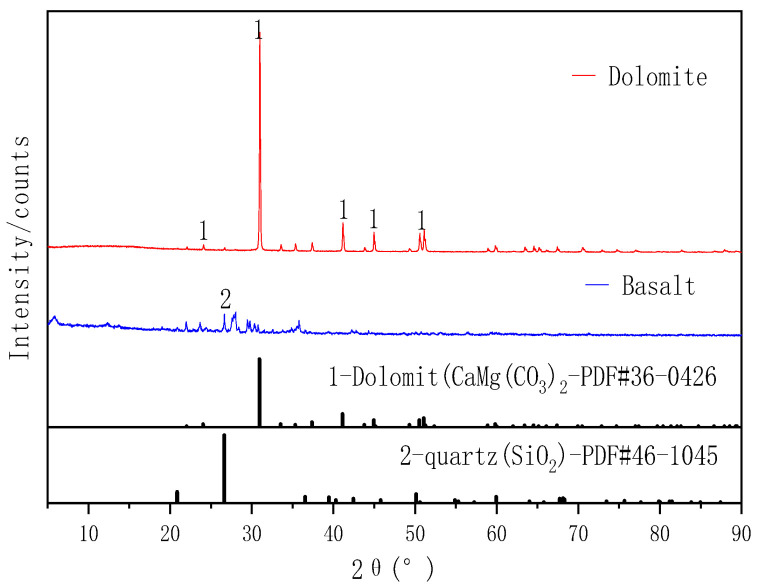
XRD patterns of the aggregates (1: dolomite (CaMg(CO_3_)_2_), 2: quartz (SiO_2_)).

Basalt and dolomite were used as a silicate aggregate and carbonate aggregate, respectively. Both were taken from a sand field in Guiyang, China. The aggregates were broken and screened into several particle size grades. Particles sized 1–3 mm were taken out, washed with laboratory neutral tap water, and then placed in an oven at 60 °C for 4–5 h. Then, the samples were dried to a constant weight. XRD (Figure 3) showed that the main minerals in the basalt and dolomite aggregates were dolomite and quartz, respectively.

### 2.2. Mix Proportions

According to the Chinese national standard GB/T17671-1999, which is used to test the mechanical properties of cementitious materials, the cementitious materials were 450 g, the water–binder ratio was 0.50, and the aggregates were 1350 g. Initially, ordinary Portland cement was used as the cementitious material, standard sands were used as the aggregates, and the water was laboratory tap water. Then, the cement was replaced with RM. The proportions were 0 wt.%, 3 wt.%, 10 wt.%, and 40 wt.%, respectively. Table 2 shows the concrete’s mix design proportions. Next, the optimum proportion of RM obtained above was mixed at YPS levels of 0 wt.%, 9 wt.%, 18wt.%, and 63 wt.%, respectively (Table 3). Through the above tests, we obtained the best ratio of RM and YPS substituted for cement when preparing concrete. 

Finally, basalt was used as a silicate aggregate and dolomite was used as a carbonate aggregate. RM–YPS–cement (at a mixing ratio ensuring the best mechanical properties of concrete) was used as a cementitious material to prepare concrete (Table 4).

### 2.3. Specimen Preparation and Experimental Methods

#### 2.3.1. Procedures of Specimen Preparation

The concrete specimens were prepared according to the Chinese national standard GBT-17671-1999 (ISO method). The processes for preparing the concrete specimen were as follows: 225 g of water and 450 g of cementitious material first were put into a blender (JJ-5, Cangzhou Kexing Instrument Equipment Manufacturing Company, Cangzhou City, Hebei Province, China). The blender contained mortar and was installed on the machine and raised to a fixed height. Then, the machine was started, and the contents were stirred for 30 s at a low speed. During the next 30 s, aggregates with a certain size were uniformly added to the container. The machine stopped at 90 s after stirring for 60 s at a high speed. Meanwhile, we scraped the mortar stuck to the propeller wall 15 s after stopping. We restarted the machine and stopped it after stirring for 60 s at a high speed. Next, we put the 40 mm × 40 mm × 160 mm mold on a shaking table. The mold was used to prepare the concrete with a fixed shape. We filled the mold half-full evenly via a prepared mortar. We vibrated it for 60 s ± 5 s. Then, we filled the mold and vibrated it again for 60 s ± 5 s. We removed the mold from the shaking table and scraped spilled mortar from the edge and surface of the mold until it was flattened. Finally, we took out the concrete from the mold and put it into the curing box to be cured at room temperature for 24 h. Special attention was paid to keeping the curing box at a temperature of 20 ± 1 °C and a relative humidity of at least 90%.

#### 2.3.2. Measurement of Mechanical Performance

The flexural strength and compressive strength, which reflect the mechanical performance of the specimens, were measured via microcomputer-controlled compression and a bending tester (YAW-3000B, Zhejiang Yingsong Instrument Equipment Manufacturing Company, Shaoxing City, Zhejiang Province, China). We put the specimen on the machine’s support frame and kept its long axis perpendicular to the support frame. Then, we applied pressure at a constant rate of 50 N/s to the side relative to the support surface until it broke. We then calculated the bending strength R_f_ (MPa) of the specimen using Equation (2).

R_f_ = 1.5 × F_f_ × L/b^3^ (2)

where F_f_ is the load applied to the middle of the specimen when it is broken (N), L is the distance of the cylindrical frames (±5 mm), and b is the side length of the square section of the specimen.

We aligned the center of the broken part obtained from the flexural strength test with the center of the support platform, where the end of the specimen was about 10 mm beyond the edge of the support platform. We applied pressure at a constant rate of 0.5 KN/s until it was destroyed. We calculated the compressive strength Rc (MPa) of the specimen using Equation (3).

Rc = Fc/A(3)

where Fc is the maximum load (N) when the specimen is broken and A is the area of the compressed part (mm^2^, 40 mm × 40 mm = 160 mm^2^).

#### 2.3.3. Microporous Structure Measurement of ITZ

The distribution of the pore size and pore volume of the ITZ in concrete were studied via the mercury intrusion porosimetry method (MIP) using a high-performance automatic mercury porosimeter (9510, Micromeritics Instrument Corporation, Atlanta, GA, USA). The pore size range was measured to be between 5 nm and 340,000 nm. The specimens damaged after the test described in 2.3.2 were further broken into sizes of 5–9 mm. To prevent further hydration in the concrete, the specimens were soaked in anhydrous ethanol for 24 h and then dried in the oven to a constant weight (for about 4 h, at 60 °C). Particles containing both matrices and aggregates were selected for testing. The mercury was pressed into the hole of specimens and the relationship between the pressure (p) of mercury injected into hole and the hole radius R (nm) is as follows.

R = 2 × σ × cosβ/p(4)

where σ is the surface tension of mercury, β is the wetting angle between the specimen and mercury, and p is the injection pressure. The hole radius could be obtained according to the p, and then the size and total area of the hole were calculated.

#### 2.3.4. Measurement of Structure and Morphology of ITZ

The ITZ morphology and structure in the concrete were studied using a scanning electron microscope (SEM) (Zeiss Sigma 300, ZEISS Company, Jena, Thuringia, Germany). According to the test requirements, the plastic cylinders with inner diameters of 10 mm and length of 30 mm were self-made. Its bottom was sealed with a plastic sheet. The composite cementitious material (or pure cement) and water were well mixed at 2:1 to produce paste. The obtained paste was added up to the one-third line of the cylinder; then, the aggregate of a 3–5 mm particle size was introduced. Finally, the remaining space was filled with the paste. The mold was put into the curing box (temperature, 20 ± 1 °C; humidity, at least 90%) for 24 h. The specimen was then removed from the mold and put it into the curing box with the same environment for 28 days. The specimen was removed and detached from the part containing the aggregate and cementitious material. The broken specimens were soaked in anhydrous ethanol for 24 h to stop hydration. They were then moved to the drying oven for incubation at 60 °C for about 4 h to dry to a constant weight. The fracture surface of the specimen was gilded to prevent an electric charge for the test results. We observed the morphology and structure of the ITZ between aggregate and cementitious material via SEM.

#### 2.3.5. Measurement of Interfacial Material Composition

Dolomite and basalt aggregates were finely ground to −0.075 mm grains. The aggregate powder was mixed with composite cementitious material powder or pure cement powder at a 1:1 ratio. It mixed with water at 1:2, stirred well, and then put into a curing box for 28 days. The specimen was moved to the oven to dry to a constant weight. The specimen was ground again to −0.075 mm, and the mineral composition of the ITZ was tested via XRD (X PertPowder, Panako X-ray Analysis Instruments, Almelo, Overijssel, Netherlands).

## 3. Results and Discussion

### 3.1. Effect of Replacing Cement with YPS and RM on the Mechanical Properties of Concrete

#### 3.1.1. Effect of Replacing Cement with RM on the Mechanical Properties of Concrete

**Figure 4:**
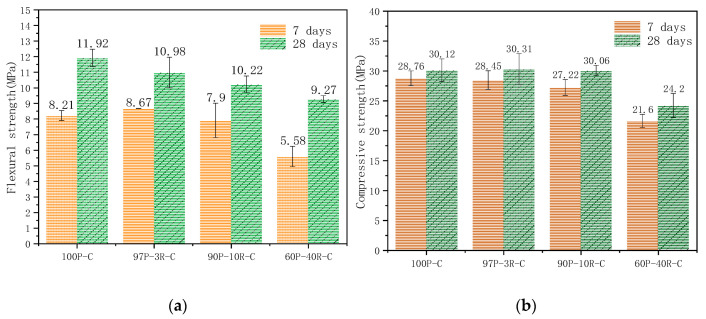
The curve of (**a**) flexural strength and (**b**) compressive strength of concrete prepared with RM partially substituting ordinary Portland cement.

Mechanical properties are the most important and basic performance parameters for engineering quality when applying concrete. The mineral composition of RM is complex. Although the content of aluminosilicate minerals is high, the properties of silicon-oxygen bonds and aluminum-oxygen bonds are stable and difficult to release. Therefore, the low activity of RM means that it cannot completely replace cement. The effects of replacing cement with RM with additive amounts of 0 wt.%, 3 wt.%, 10 wt.%, and 40 wt.% on the mechanical properties of concrete were studied. The proportions of the admixture are shown in Table 2, and the test results are shown in Figure 4.

We found a strong correlation between RM and the mechanical properties of concrete. The trends of change in the flexural strength and compressive strength are similar after curing for 7 days and curing for 28 days. Generally, the mechanical properties of the concrete decreased when the RM increased. RM not only had no effect but also improved the compressive strength of concrete when the addition was less than 3 wt.%. However, the correlation between RM when more than 3 wt.% was added and the mechanical properties of the concrete was significantly negative. Many scholars have confirmed this finding [36,37]. This is because RM particles could fill micropores and optimize the microstructure of the concrete [38]. However, Bayer RM had a weak gelling property, and the cementitious substances in the matrix and ITZ were diluted when the ratio of Bayer RM was large, as it reduced the mechanical properties of the concrete. Therefore, not too much RM was added, and the effect on the mechanical properties of the concrete of a low content of RM was little. Shown in Figure 4b, compared with ordinary Portland cement concrete (100P-C), the change in the concrete’s compressive strength with 10 wt.% RM (90P–10R–C) was little and gradually decreased.

#### 3.1.2. Effect of Replacing Cement with YPS–RM on Mechanical Properties of Concrete

**Figure 5:**
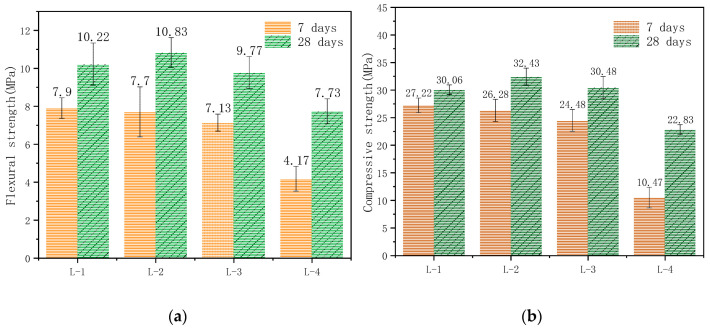
The curve of (**a**) flexural strength and (**b**) compressive strength for concrete prepared with RM and YPS partially substituting ordinary Portland cement.

The comprehensive utilization of YPS involves many fields, but the total utilization rate is not high and much of the abundant YPS is difficult to use. YPS as a concrete admixture has some adverse effects including extending the setting time and reducing the early strength, which restricts the application of YPS in concrete. Therefore, the gelling property of YPS must be fully activated when increasing the YPS content in concrete. Previous studies have shown that YPS can refine the later pore structure of hardened paste, reduce the chloride ion diffusion coefficient, and improve the compressive strength and concrete durability [39]. Additionally, the activity of clinker minerals in the dissolution process improved when YPS fluorine (F) and phosphorus (P) were added. These materials are used for improving the strength of cement clinker [40]. Chen et al. [41] found that ordinary Portland cement can be partially or completely replaced by YPS without affecting the potential coagulation performance, according to a study on the cemented filling performance of YPS. Abundant alkaline substances and certain pozzolanic active substances in RM are able to simulate the gelling active substances in YPS and generate a synergistic effect. The effect of the YPS content on concrete’s mechanical properties was studied under the condition of 10 wt.% RM being added. The ratio of the admixture is shown in Table 3, and the test results are shown in Figure 5.

Figure 5 shows the coincidental change in the relationships for the flexural strength and compressive strength in concrete after concrete was prepared with YPS and RM and cured for 7 days and 28 days. The mechanical properties of concrete slightly improved when 10 wt.% RM and 9 wt.% (L-2) YPS were added for 28 days. When the YPS was over 18 wt.%, the flexural or compressive strength decreased quickly. Furthermore, the flexural strength of concrete with 10 wt.% RM and 63 wt.% YPS (L-4) cured for 7 days and 28 days decreased by 47.22% and 24.36%, and the compressive strength decreased by 61.54% and 24.05%, respectively. Therefore, it is necessary to use YPS in combination with other substances to obtain a good gelling property due to the poor activity of YPS.

A good gelling property cannot be obtained when using YPS alone as a cementitious material. The compressive strength related to the internal structure of hardened cement paste directly reflected the quality of concrete. This is a significant parameter for the mechanical properties of concrete. Shown in Figure 5b, the slope of the compressive strength curve for concrete with cement replacement (YPS of 0.00 wt.% (L-1) to 63.00 wt.% (L-4) for 7 days) was higher than that for the 28-day-cured concrete, mainly due to the large amount of P in YPS. The results showed that P could slow down the hydration of ordinary Portland cement and extend the concrete’s plasticity. Additionally, with the increase in YPS, the degradation of concrete was more obvious. The flexural and compressive strength of the concrete with a 9.00 wt.% YPS content were higher than those of the concrete with non-phosphorus slag. Using 18.00 wt.% YPS, the mechanical properties began to weaken and the flexural compressive strength of the concrete with YPS was close to that of concrete without YPS. Therefore, the effects of a 10.00 wt.% RM content and low YPS content on the mechanical properties of concrete were slight. The main reason for the improvement in the mechanical properties of the concrete was the minor addition of YPS, whose cementitious activity via active SiO_2_ and a small amount of active Al_2_O_3_ in the YPS generated calcium silicate hydrate (CSH) gel and calcium aluminate hydrate (CAH) gel. Additionally, the non-active particles in YPS and RM filled the matrix and ITZ, improving the internal structure and mechanical properties of the concrete. The basicity of the paste weakened with a large amount of YPS; meanwhile, the cementitious activity of active silicon and aluminum in the YPS did not lead to low mechanical properties for the concrete.

### 3.2. Effect of Aggregates on Mechanical Properties of Concrete Prepared with RM–YPS–Cement

**Figure 6:**
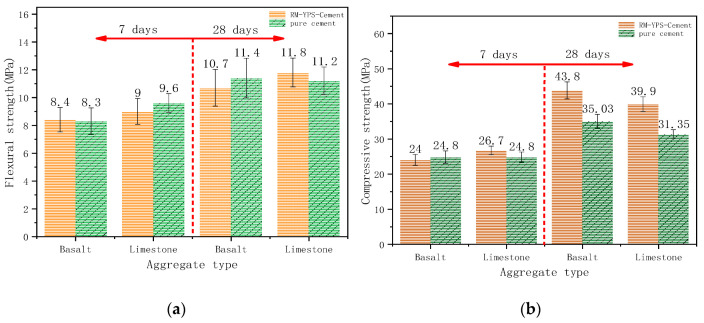
(**a**) Flexural strength and (**b**) compressive strength of concrete with different aggregates and RM–YPS–cement.

The aggregate is a skeleton support in concrete and reduces the effect of the dry shrinkage and moisture expansion of cementitious materials in the process of setting and hardening. Aggregates are divided into silicate aggregate and carbonate aggregate for mineral composition in industrial applications. The ITZs formed by different aggregates and cementitious materials have different microstructures, which are reflected in the mechanical properties of the concrete. During this study, basalt was used as the silicate aggregate and dolomite was used as the carbonate aggregate. We then studied the adaptability of different aggregates in composite cementitious materials (RM–YPS–cement). The strength test results of concrete prepared with the RM–YPS–cement (10.00 wt.% RM, 18.00 wt.% YPS, 72 wt.% cement) as the cementitious material with dolomite and basalt as the aggregate, respectively, were shown in Figure 6. There was no significant difference in flexural strength (Figure 6a) in the concrete prepared with the RM–YPS–cement. The difference in the concrete’s compressive strength (cured for 7 days) was not obvious (Figure 6b). The reason may be that the P element in YPS led to the degradation of the concrete and affected the improvement of the concrete’s compressive strength. The concrete’s compressive strength (cured for 28 days and prepared with RM–YPS–cement) was much higher than that of concrete prepared with pure cement as the cementitious material (basalt and dolomite aggregates increased it by 25.04% and 27.27%, respectively). Therefore, the mechanical properties of concrete were improved by replacing cement with certain proportions of RM and YPS.

### 3.3. Microporous Structure in ITZ

**Figure 7:**
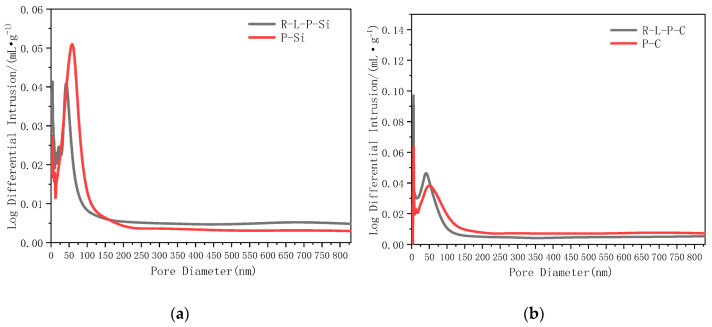
Pore size distribution of ITZ in concrete prepared with RM–YPS–cement and pure cement after 28 days of curing ((**a**): Pore size distribution of ITZ of R–L–P–Si and P–Si concrete; (**b**): Pore size distribution of ITZ of R–L–P–C and P–C concrete).

Abundant micropores with different sizes exist in concrete, which have different effects on concrete properties (those below 20 nm are harmless pores, those with 20–100 nm diameters are less harmful pores, and those with diameters over 100 nm are harmful pores [42]). The pore size and distribution in the ITZ in concrete prepared with RM–YPS–cement and pure cement combined with basalt and dolomite aggregates, after curing for 28 and 7 days, are shown in Table 5 and Figure 7.

Compared to the concrete prepared with pure cement and the basalt aggregate, the intermediate pore diameter of the concrete prepared with RM–YPS–cement and the basalt aggregate decreased by 32.28%. The average pore diameter decreased by 23.37%. However, the total pore area increased by 41.78%. Shown in Figure 7, the pore quantity (10–50 nm) in the ITZ in the concrete prepared with RM–YPS–cement was more than that in the concrete prepared with pure cement, but the quantity of pores over 50 nm was lower, regardless of whether the basalt or dolomite aggregate was used. This was because micropores in the ITZ region are filled by nonreactive YPS and RM particles. Since small RM particles resulted in large pores and were divided into many small pores, the average pore diameter decreased, yet the total pore area increased. Additionally, the cementitious activity of YPS was activated using cement hydration products. The active SiO_2_ reacted with CH in ITZ to form CSH and filled interfacial micropores, which promoted cement hydration. Moreover, the RM with low cementitious materials and a small particle size could absorb excess water to prevent the formation of abundant harmful pores after water evaporation. They also could divide large pores into many small pores and decrease the average pore diameter while increasing the total pore area. Therefore, a certain proportion of RM and YPS can decrease the average pore size and promote the production of 10–50 nm micropores in concrete. A large number of harmful pores were transformed into harmless pores and less harmful pores, resulting in the mechanical properties of concrete being greatly improved.

### 3.4. ITZ Structure and Topography

**Figure 8:**
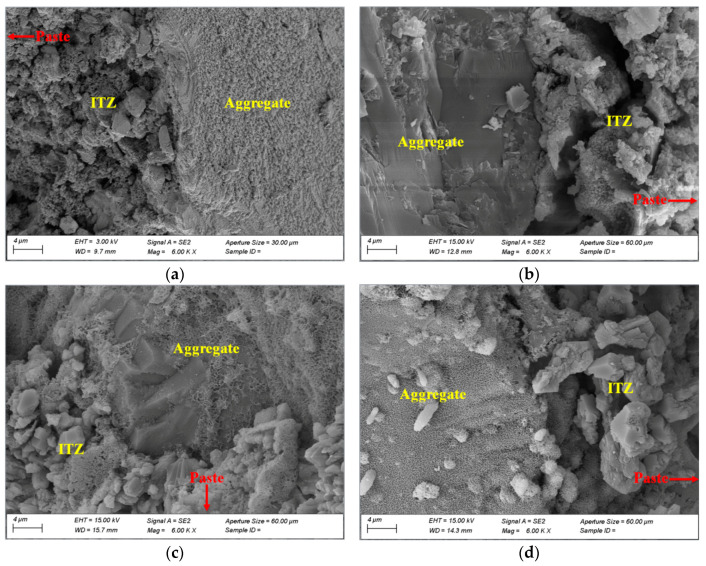
SEM test results for ITZ in concrete prepared with pure cement and RM–YPS–cement with dolomite and basalt aggregates, respectively ((**a**): RM–YPS–cement–basalt aggregate; (**b**): pure cement-basalt aggregate; (**c**): RM–YPS–cement–dolomite aggregate; (**d**): pure cement–dolomite aggregate).

The ITZ is the transition zone between the aggregate and the matrix, the thickness of which is 40–100 μm from the aggregate interface. The ITZ is the weakest link of the concrete [43]. Additionally, the crack width, pore size, and bond degree of the ITZ directly determine the mechanical properties of concrete [44,45,46]. The SEM testing results for the ITZ in the concrete prepared with RM–YPS–cement (RM:YPS:cement = 10:18:72) and pure cement after curing for 28 days are shown in Figure 8. The results show that the concrete prepared with RM–YPS–cement and dolomite or basalt aggregate had a smaller interface pore diameter, a more compact ITZ, and a narrower microcrack, which are consistent with the test results for the ITZ pore size distribution. The interface between aggregates and cementitious materials has a great influence on the mechanical properties of concrete [47]. Therefore, replacing the cement with RM and YPS can optimize the interfacial structure, promote force conduction, and improve the mechanical properties. According to Figure 8, the crystal size of the ITZ in concrete prepared with RM–YPS–cement is smaller and adheres closely to the aggregate surface, which benefits the occlusion between the cementitious materials and aggregates. Due to the low activity and small particle size of RM, the micropores of the matrix and interface filled. Furthermore, a large amount of highly active SiO_2_ in the YPS participated in the reaction, leading to the optimization of the interfacial structure.

### 3.5. Mineral Composition of ITZ

**Figure 9:**
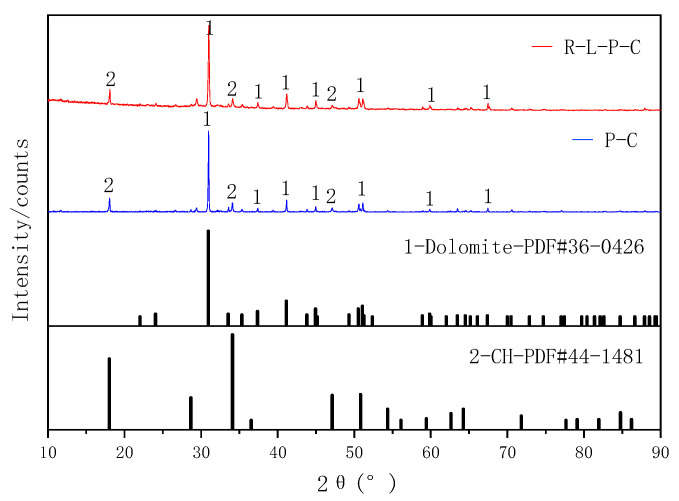
XRD patterns of the reaction product between dolomite aggregate and cement materials (1: dolomite (CaMg(CO_3_)_2_), 2: CH (Ca(OH)_2_)).

**Figure 10:**
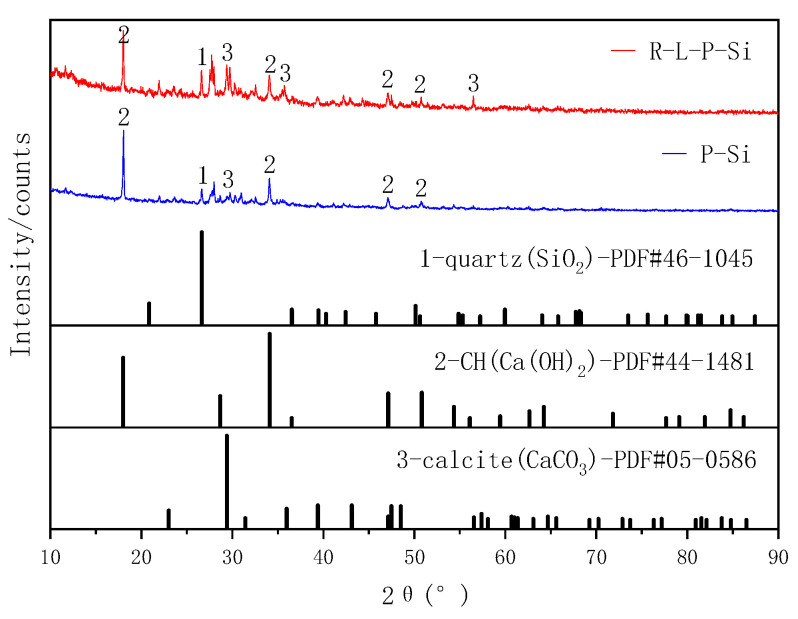
XRD patterns of the reaction product between basalt aggregate and cement materials (1: quartz ((SiO_2_), 2: CH (Ca(OH)_2_), 3: calcite (CaCO_3_)).

XRD was used to test the material composition resulting from the reaction of dolomite and basalt aggregates with RM–YPS–cement and cement, respectively (the solid–liquid ratio was 1:1). We studied the effect of RM and YPS on the material composition of the ITZ in concrete. Shown in Figure 9 and Figure 10 (the sample numbers are R–L–P–C (concrete with RM–YPS–cement and carbonate aggregate), P–C (concrete with ordinary Portland cement and carbonate aggregate), R–L–P–Si (concrete with RM–YPS–cement and silicate aggregate), and P–Si (concrete with ordinary Portland cement and silicate aggregate)), the mineral composition of the reaction products for the dolomite aggregate with RM–YPS–cement and cement showed no difference. This indicates that the RM–YPS–cement does not form new mineral phases in the ITZ. Regarding the basalt aggregate, this conclusion is consistent with that for the dolomite aggregate. It is worth noting that the enhancement of the calcium carbonate peak was mainly due to the introduction of RM calcite minerals. Whether using dolomite or basalt as the aggregate, the addition of RM and YPS in the concrete weakened the peak of CH in the reaction product, mainly because the abundant amorphous silica in YPS reacted with CH to form CSH gel (the reaction formula is shown in (5)). It also filled the porous area in the ITZ and reduced the pore size, which could improve the mechanical properties of concrete. Additionally, CH in the matrix also reacts with amorphous silica in YPS to promote cement hydration (the reaction formulas are shown in (6)–(9)).

Amorphous SiO_2_ + Ca(OH)_2_⟶CSH +H_2_O (5)

2(3CaO·SiO_2_) + 6H_2_O ⟶ 3CaO·2SiO_2_·3H_2_O + 3Ca(OH)_2_ (6)

2(2CaO·SiO_2_) + 4H_2_O ⟶ 3CaO·2SiO_2_·3H_2_O + Ca(OH)_2_ (7)

3CaO·Al_2_O_3_ + 6H_2_O ⟶ 3CaO·Al_2_O_3_·6H_2_O (8)

4CaO·Al_2_O_3_·Fe_2_O_3_ + 2Ca(OH)_2_ + 10H_2_O ⟶ 3CaO·Al_2_O_3_·6H_2_O + 3CaO·Fe_2_O_3_·6H_2_O (9)

## 4. Conclusions

We found a slight decrease in the concrete compressive strength when adding 10.00 wt.% of RM; when we added more than 10.00 wt.%, it decreased greatly. The mechanical properties of concrete for which cement was replaced with 10 wt.% RM and 9.00 wt.% YPS were better than when it was replaced with only 10 wt.% RM and 18.00 wt.% YPS. Regarding the basalt and dolomite aggregates, the concrete compressive strength of the RM–YPS–cement increased by 25.04% and 27.27%, respectively, at the curing age of 28 days.

The crystal size of the ITZ in the concrete prepared with RM–YPS–cement was smaller, and the crystals adhered closely to the aggregate surface, which was beneficial for an effective occlusion between the cementitious materials and aggregates. Nonreactive YPS and RM particles could fill micropores in the ITZ region. Meanwhile, the small particle size of RM and YPS resulted in large pores being divided into many small pores, which decreased the average pore diameter. Adding RM–YPS–cement increased the pore diameter in the ITZ; the pores were mostly 10–50 nm, and more compact ITZ structures and narrower microcracks were formed.

Although the cementitious material added 10.00 wt.% RM and 18.00 wt.% YPS, it did not form new mineral phases in the ITZ. Instead, it led to a decrease in CH in the reaction product, mainly because a large amount of amorphous silica in the YPS reacted with CH to form CSH gel. This resulted in the ITZ porous area filling and a reduction in pore size, which can improve the mechanical properties of concrete. Additionally, CH in the matrix also reacted with amorphous silica in YPS, promoting cement hydration.

## Figures and Tables

**Table 1:** The main chemical composition of the raw materials.

Name	Oxide Composition (wt.%)								
	SiO_2_	CaO	Al_2_O_3_	TiO_2_	Fe_2_O_3_	MgO	K_2_O	Na_2_O	P_2_O_5_
RM	17.33	16.32	21.09	4.69	21.93	1.89	1.21	1.46	0.34
YPS	37.22	44.77	5.42	0.26	0.45	2.61	1.43	0.33	2.83
Cement	20.08	60.65	4.61	0.55	3.36	1.98	0.54	0.85	0.22

**Table 2:** The mix design proportions of concrete prepared with RM (by weight).

No.	Cement (%)	RM (%)	Cementitious Material (g)	Aggregate (g)	Water (mL)
100P-C	100.00	0.00	450.00	1350	225
97P-3R-C	97.00	3.00			
90P-10R-C	90.00	10.00			
60P-40R-C	60.00	40.00			

**Table 3:** The mix design proportions of concrete prepared with RM and YPS (by weight).

No.	RM (g)	YPS (g)	Cement (g)	Aggregate (g)	Water (mL)
L-1	45.00	0.00	405.00	1350	225
L-2		40.50	364.50		
L-3		81.00	324.00		
L-4		283.50	121.50		

**Table 4:** The mix design proportions of concrete prepared with RM, YPS, and different aggregates (by weight).

No.	RM (g)	YPS (g)	Cement (g)	Aggregate Type	Aggregate (g)	Water (mL)
A-1	45.00	81.00	324.00	dolomite	1350	225
A-2				basalt		

**Table 5:** Test results for the ITZ pore size in concrete prepared with RM–YPS–cement and pure cement after 28 days of curing.

Aggregate Type	Silicate Aggregate		Carbonate Aggregate	
Cementitious Material	Pure Cement	RM–YPS–Cement	Pure Cement	RM–YPS–Cement
Concrete No.	P–Si	R–L–P–Si	P–C	R–L–P–C
Intermediate aperture/nm	50.5	34.2	42.5	27.3
Total pore area/m^2^/g	10.978	15.565	14.054	17.894
Average aperture/nm	18.4	14.1	17.0	14.4
Voidage/%	11.9965	12.9124	13.8027	14.7358

## Data Availability

Data available in a publicly accessible repository.
